# Analysis of aided thresholds in children who have undergone cochlear reimplantation: a ten-year follow-up

**DOI:** 10.1590/2317-1782/20232021293en

**Published:** 2023-10-30

**Authors:** Lucas Bevilacqua Alves da Costa, Leticia Cristina Vicente, Leandra Tabanez do Nascimento Silva, Kátia Freitas Alvarenga, Manoel Henrique Salgado, Orozimbo Alves Costa, Rubens Brito

**Affiliations:** 1 Alfa Instituto de Comunicação e Audição - São Paulo (SP), Brasil.; 2 Centro Universitário Planalto do Distrito Federal - Brasília (DF), Brasil.; 3 Seção de Implante Coclear, Hospital de Reabilitação de Anomalias Craniofaciais (HRAC) - Bauru (SP), Brasil.; 4 Departamento de Fonoaudiologia, Faculdade de Odontologia - FOB, Universidade de São Paulo - USP - Bauru (SP), Brasil.; 5 Departamento de Engenharia de Produção, Faculdade de Engenharia de Bauru, Universidade Estadual Paulista - UNESP - Bauru (SP), Brasil.; 6 Departamento de Otorrinolaringologia, Faculdade de Medicina, Universidade de São Paulo - USP - São Paulo (SP), Brasil.

**Keywords:** Children, Reimplantation, Cochlear Implant, Hearing, Audiometry, Pure-Tone

## Abstract

**Purpose:**

To characterize hearing thresholds at frequencies of 500, 1000, 2000 and 4000 Hz in children undergoing reimplantation with a follow-up of at least 10 years.

**Methods:**

Retrospective review of medical records of children who underwent reimplantation surgery for at least 10 years. The auditory thresholds obtained in free-field pure tone audiometry with the cochlear implant were evaluated at frequencies of 500, 1000, 2000 and 4000 Hz at four different times: 1 (before failure), 2 (activation), 3 (five years after reimplantation) and 4 (ten years after reimplantation, regardless of the time of use of the 2nd CI) in patients with a follow-up of at least 10 years.

**Results:**

Evaluating patients who underwent reimplantation, it was observed that the thresholds of 500, 1000, 2000, 4000 Hz were similar in the long term to those obtained in patients who were implanted only once, thus not presenting damage in the detection of sounds.

**Conclusion:**

Reimplantation had no long-term effect on the hearing thresholds obtained in children who underwent this surgery due to internal component failure.

## INTRODUCTION

Cochlear implants have been implanted routinely in approximately 500,000 individuals worldwide. The surgical procedure is considered safe and successful for the treatment of severe and deep hearing loss^(^ 1 ^)^. Thus, these implants are the main route for hearing rehabilitation and habilitation in such cases, allowing children to develop hearing and language, in addition to better school performance and improved perspectives regarding their future entering the work market^(^ 2-4 ^)^. For post-lingual adults, the cochlear implant can reestablish communication and improve life quality^(^ 5-7 ^)^.

The first surgeries of multichannel cochlear implants were performed in the 90s, naturally representing a concern among professionals of the area whether the internal component will cease working properly due to wear, hence requiring another surgical intervention. Studies have demonstrated that the rate of surgical review of the cochlear implant is between 5 and 10%^(^ 1 ^)^, whose most frequent cause is repeated device failure, thus demanding reimplantation^(^ 8 ^)^.

In addition to the risks inherent to surgical intervention, reimplantation implies a particular concern with the impact on hearing performance and speech-hearing perception, that is, whether the patient will maintain the benefit obtained from the first surgery. Such an issue is linked to the potential difficulty of the surgeon when inserting a new electrode in the cochlea, which might follow a different route from the old electrode, hence stimulating other parts of the cochlea^(^ 9 ^)^. Furthermore, the cochlea might be ossified, hindering the removal of the old electrode or making the removal only partial, and affecting the patient’s hearing performance^(^ 9 ^)^.

The analysis of the results obtained after the reimplantation surgery by distinct causes has contributed to enlarging the knowledge of the consequences of another surgical intervention^(^ 10 ^)^. The causes reported for reimplantation include ‘hard failure,’ which is characterized by a sudden stop in the functioning of the internal component of the cochlear implant, as well as ‘soft failure,’ which is a drop in the patient’s hearing performance. Such a decrease is often progressive and confused with child developmental issues that might be linked to comorbidities and cognitive matters. This failure is hard to detect and, in many cases, there are changes in impedance telemetry and neuro-telemetry^(^ 11-13 ^)^. Hard failure is described as the most common cause of reimplantation^(^ 14-18 ^)^.

In general, reimplantation is considered a safe procedure with good hearing results in most patients^(^ 9,14,15,17-19 ^)^. However, there are no long-term studies describing whether the results found in children linked to the hearing thresholds remain many years after reimplantation. Such information is fundamental since hearing attention and detection are hearing skills required for the development of more complex skills, such as speech perception.

Hearing attention and detection are the first hearing skills to be acquired by children, thus being fundamental for the developmental process of all other skills. Thereby, this study aimed to characterize the hearing thresholds along 10 years in children subjected to cochlear reimplantation.

## METHOD

This study was approved by the Ethics Research Committee of the Craniofacial Anomalies Rehabilitation Hospital of the University of São Paulo (HRAC-USP), protocol number 673836/2014. This is a retrospective, long-term study based on the analysis of the medical history of individuals treated at the Section of Cochlear Implant at the Hearing Research Center of the HRAC-USP. From a total of 1323 cochlear implant surgeries performed from 1990 to January 2016, 84 cochlear reimplantation surgeries were registered, corresponding to 6.3% of the total.

We selected data from 12 users of unilateral cochlear implants presenting hard failure in the internal device Combi 40+ by MED-EL and subjected to cochlear reimplantation with another internal device Combi 40+ in the same ear. All participants presented total insertion of electrodes and had their hearing skills monitored before the failure and for ten years after the reimplantation surgery. In addition, they had no neurological and/or cognitive impairments. Table 1 shows the participants’ ages at the first cochlear implants (CI) surgery and reimplantation, as well as the time of re-approach.

**Table 1:** Population Data

Child	Etiology	Age 1^st^ surgery (months)	CI usage time at device failure (months)	Implanted Ear	Re-approach time (months)
1	Cytomegalovirus	32	19	RE	2
2	Idiopathic	45	25	RE	3
3	Rubeola	89	66	LE	1
4	Idiopathic	46	37	LE	4
5	Idiopathic	37	22	LE	4
6	Idiopathic	41	20	LE	2
7	Ototoxic	55	46	RE	5
8	Rubeola	84	44	RE	2
9	Meningitis	62	30	RE	3
10	Meningitis	24	31	LE	4
11	Idiopathic	40	46	LE	1
12	Meningitis	36	32	RE	5
Mean ± SD	-	49.25±20.06	34.83±13.78		3±1.41
Months		(32-89)	(19-66)		(1-5)

Caption: SD = Standard deviation; CI = Cochlear Implant; RE = Right Ear; LE = Left Ear

### Procedures

The hearing thresholds obtained in free-field pure tone audiometry were assessed only with the use of the cochlear implant at the frequencies of 500, 1000, 2000, and 4000 Hz in four distinct moments: 1 (before the failure), 2 (activation), 3 (five years after reimplantation), and 4 (ten years after reimplantation, regardless of the usage time for the 2^nd^ CI).

### Data analysis

The descriptive statistical analysis of the hearing thresholds was performed considering the tested frequencies at 500, 1000, 2000, and 4000 Hz for the four assessment moments.

The correlations between the tested frequencies and assessment moments were performed using a Two-way ANOVA. The post hoc tests - Tukey method -calculated the significant differences between the assessment moments. Statistical significance was set at the 5% level.

## RESULTS

Table 2 provides information on the mean, range, standard deviation, and median of the hearing thresholds at 500, 1000, 2000, and 4000 Hz measured before the device failure in the activation of the reimplanted device and five and ten years after reimplantation.

**Table 2:** Describing the hearing thresholds obtained at the frequencies of 500, 1000, 2000, and 4000 Hz from the assessments 1 (before the failure), 2 (activation), 3 (five years after the reimplantation), and 4 (ten years after the reimplantation)

**Frequency (Hz)**	**Assessment moments**	**Hearing thresholds (dB HL)**				
		**Mean**	**SD**	**Min**	**Max**	**Median**
500	Before the failure	33.33	4.92	25.00	40.00	35.00
	Activation	37.92	9.64	30.00	65.00	35.00
	5 years later	23.33	4.92	20.00	35.00	20.00
	10 years later	24.17	5.15	20.00	35.00	22.50
						
1000	Before the failure	35.83	4.69	30.00	45.00	35.00
	Activation	37.92	9.16	25.00	60.00	35.00
	5 years later	24.58	4.50	20.00	30.00	25.00
	10 years later	25.00	4.77	20.00	35.00	25.00
						
2000	Before the failure	33.75	5.28	25.00	40.00	35.00
	Activation	41.67	11.15	30.00	60.00	40.00
	5 years later	23.75	4.33	20.00	30.00	22.50
	10 years later	24.58	6.20	20.00	40.00	22.50
						
4000	Before the failure	35.42	6.89	25.00	45.00	35.00
	Activation	42.08	12.33	30.00	65.00	37.50
	5 years later	26.25	5.28	20.00	35.00	27.50
	10 years later	27.92	6.20	20.00	35.00	27.50

Caption: SD = Standard deviation; Min = Minimum; Max = Maximum

**Figure 1:**
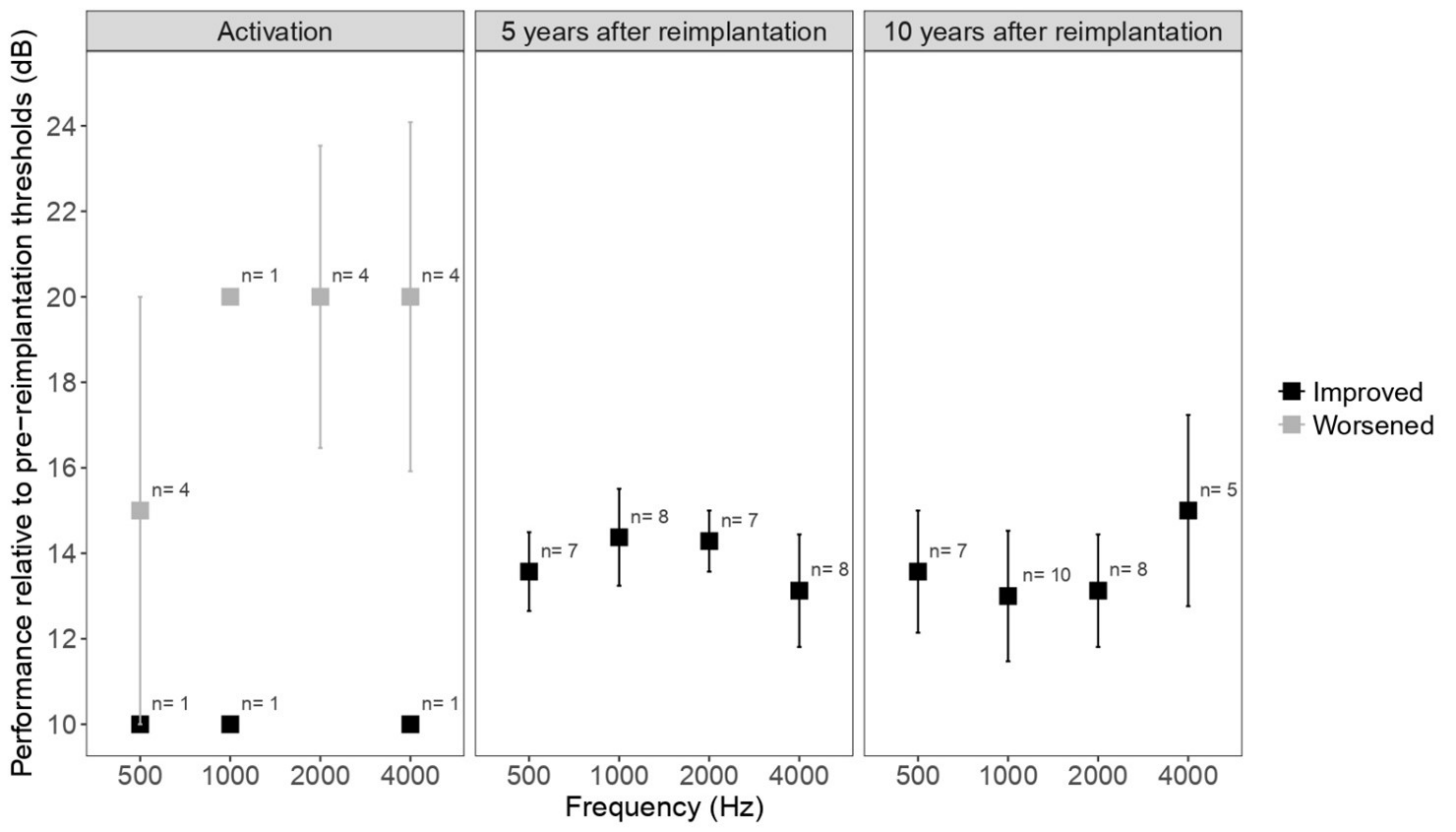
Mean ± standard error (SE) for the threshold differences considered either an improvement or a worsening for 500, 1000, 2000, and 4000 Hz plotted for each comparison before and after the cochlear reimplantation. The number of children considered for each value is presented above as the mean symbol. The results for the other children were not considered in this chart since their thresholds remained after reimplantation, hence the difference was 0 or 5 dB

Comparisons between the thresholds measured before the CI failure and the following sessions revealed that despite some children showing worse thresholds right after the activation, all thresholds reached five and ten years were either maintained or improved. Figure 1 shows the difference in the thresholds between the sessions before and after the reimplantation considered as either improvement or worsening.

**Figure 2:**
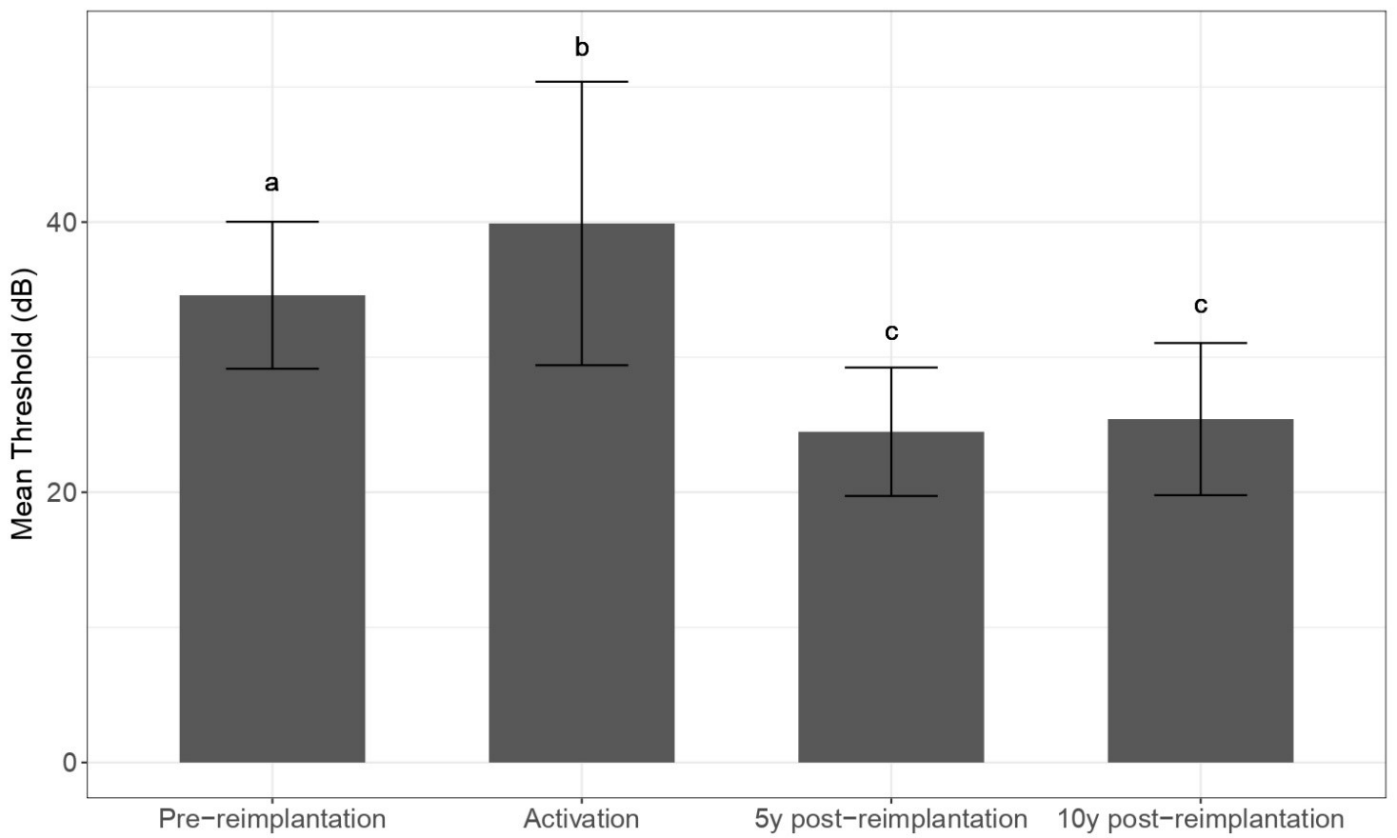
Mean ± standard error (SE) at all tested frequencies (500, 1000, 2000, and 4000 Hz) set for each measurement time. Different letters above the bars indicate significant differences in the Tukey test (p <0.05)

Two-way ANOVA for repeated measures compared the hearing thresholds according to the frequency and measure time. The main effect is shown only for the measured time, without significant interaction with the tested frequencies (Table 3). The means of the thresholds for each frequency were calculated on average for post-hoc analysis. A post-hoc Tukey test showed no significant difference between the thresholds five and ten years after reimplantation. However, such thresholds are significantly better than those measured before the device failure and in the activation. The threshold values were worse in the activation than those reached before the failure (Figure 2).

**Table 3:** Comparing the hearing thresholds between the assessment moments and tested frequencies (Two-way ANOVA)

	**Frequencies**	**p**
Moments	53.54	0.000*
Frequency	1.74	0.160
Interaction	0.33	0.963

* Significance level p≤0.05

## DISCUSSION

Concerning the cochlear reimplantation, the need for removal followed by a new insertion of the arrangement of intra-cochlear electrodes raises the issue that such an intervention could compromise hearing outcomes. Seyyedi and Nadol^(^ 20 ^)^ observed the temporal findings from 21 adult users of CI. A chronic inflammatory process in the electrodes led to fibrosis and new bone formation, the inflammatory response was less intense around the electrodes closer to the more apical region of the cochlea. Such a condition is likely to make electrode insertion difficult in an eventual reimplantation. Concern about electrode insertion trauma in cochlear implantation led to the concept of soft surgery. In soft surgery, the surgical approach aims to preserve the structure of the cochlea by combining several procedures, such as the use of substances associated with hearing preservation: hyaluronic acid and corticoids, less invasive electrodes, and a longer time in the insertion of the electrode. Such practices are important not only to preserve auditory residues but also to improve surgical conditions in case of need^(^ 21-23 ^)^.

In addition to the condition of the cochlea in the reimplantation moment, it is worth considering that a new electrode array insertion does not always follow the primary path^(^ 10 ^)^. Thus, the new arrangement of electrodes might be in a different intra-cochlear space from the first insertion, allowing to raise the question of the impact of such a change on hearing performance since different parts of the cochlea can be stimulated. Clark et al.^(^ 24 ^)^, describe the trajectory of a patient who was reimplanted twice; after the patient passed away, histopathological findings of the temporal bone indicated a total loss of the organ of Corti on both sides and rupture of the basilar membrane on the left side.

In our study, the assessment of hearing thresholds in children subjected to reimplantation showed that the thresholds reached in the activation are worse than those measured before reimplantation, hence the individuals did not present the same performance in detection right after the second surgery (Figure 2). It is noteworthy that the re-approach time between the device failure and reimplantation did not exceed five months, being performed on average within three months; therefore, the patients were not hearing impaired until the moment of reimplantation. The five- and ten-year follow-ups of hearing thresholds after reimplantation showed that the immediate worse in the thresholds after activation was temporary and can be linked to a lower level of current available in the moment of activation, aiming at a greater comfort and effective use of the device. The thresholds recovered and none of the individuals showed thresholds worse than 5 dB after reimplantation regarding those detected in the last tonal audiometry before the device failure. In fact, the thresholds for five and ten years after reimplantation were even better than the previous values (Figures 1 and 2), thus demonstrating that reimplantation did not damage the development of hearing skills.

Even though frequency had no significant effect on threshold changes (Table 3), we found hearing thresholds worse than 40 dB HL in the activation in 16.66% of the children for the frequencies between 500 and 1000 Hz, while for the higher frequencies (2000 and 4000 Hz) the incidence doubled (33.33%). The electrodes of the CI are inserted to maintain the tonotopic organization of the cochlea; therefore, information on the high frequency is delivered to the electrodes in the basal region. As this region has a higher probability of surgical trauma^(^ 24 ^)^, high hearing thresholds right after reimplantation might be worse than those measured in lower frequencies. Further studies with more participants should verify such a hypothesis. However, the results obtained in five and ten years of follow-up demonstrated that the functionality of detection is maintained even for high frequencies.

Our study conducted a long-term analysis of hearing data from users of CI who were reimplanted with the same type of CI and presented the same cause for reimplantation - device hard failure. Controlling these variables allowed us to conclude that the follow-up protocol of the patients after reimplantation requires no modifications. Thereby, the same follow-up protocol must be used for the cochlear implant user without reimplantation, consisting of periodic visits for mapping, proper device maintenance, assessment of hearing and language development, and updating of the processor when necessary.

Another significant finding was that the good result for the hearing thresholds reached five years after reimplantation was maintained for the next five years (Figure 1).

The patient who needs reimplantation and their family must be informed that despite the surgery being considered a safe procedure in most cases, good results are not ensured. A lower hearing performance must be expected right after reimplantation, but the hearing thresholds will recover, and good levels will be kept until ten years after reimplantation. However, despite being a small percentage, some patients have reported not recovering the performance that they had before the reimplantation^(^ 15,25,26 ^)^.

Many studies have focused on speech perception after reimplantation^(^ 15,17,25,27,28 ^)^, which is the target skill of the CI. Nonetheless, considering the complexity of such skill and the age of the participants, herein we decided to analyze the fundamental skill to reach all others: detection. Furthermore, such skill has less influence on aspects linked to children’s development; thus, the pre-and post-intervention analyses showed that the hearing thresholds are a good parameter for the impact of reimplantation on the hearing of patients using a CI. Our findings corroborate research studies demonstrating that no statistically significant differences were found after reimplantation in 18 children between the pre- and post-reimplantation tone thresholds^(^ 29 ^)^.

## CONCLUSION

The thresholds reached at the frequencies of 500, 1000, 2000, and 4000 Hz in ten years of CI use after reimplantation showed a possible immediate worsening in the activation after the second surgery. However, these thresholds were recovered in the analysis of five years of use. The thresholds are kept stable in the following period from five to ten years of use.

## References

[1] Wick CC, Buchman CA, Ruckenstein MJ (2020). Cochlear implants and other implantable hearing devices..

[2] Niparko JK, Tobey EA, Thal DJ, Eisenberg LS, Wang N-Y, Quittner AL (2010). Spoken language development in children following cochlear implantation. JAMA.

[3] Moog JS, Geers AE (2010). Early educational placement and later language outcomes for children with cochlear implants. Otol Neurotol.

[4] Geers AE, Moog JS, Biedenstein J, Brenner C, Hayes H (2009). Spoken language scores of children using cochlear implants compared to hearing age-mates at school entry. J Deaf Stud Deaf Educ.

[5] Klop WMC, Boermans PPBM, Ferrier MB, van den Hout WB, Stiggelbout AM, Frijns JHM (2008). Clinical relevance of quality of life outcome in cochlear implantation in postlingually deafened adults. Otol Neurotol.

[6] Orabi AA, Mawman D, Al-Zoubi F, Saeed SR, Ramsden RT (2006). Cochlear implant outcomes and quality of life in the elderly: manchester experience over 13 years. Clin Otolaryngol.

[7] Damen GWJA, Beynon AJ, Krabbe PFM, Mulder JJS, Mylanus EAM (2007). Cochlear implantation and quality of life in postlingually deaf adults: long-term follow-up. Otolaryngol Head Neck Surg.

[8] Wang JT, Wang AY, Psarros C, Da Cruz M (2014). Rates of revision and device failure in cochlear implant surgery: a 30-year experience. Laryngoscope.

[9] Reis M, Boisvert I, Looi V, da Cruz M (2017). Speech recognition outcomes after cochlear reimplantation surgery. Trends Hear.

[10] Lee J, Eddington DK, Nadol JB (2011). The histopathology of revision cochlear implantation. Audiol Neurotol.

[11] Bento RF, Lima LRP, Tsuji RK, Goffi-Gomez MVS, Lima DVP, Brito R (2021). Tratado de implante coclear e próteses auditivas implantáveis..

[12] Chung D, Kim AH, Parisier S, Linstrom C, Alexiades G, Hoffman R (2010). Revision cochlear implant surgery in patients with suspected soft failures. Otol Neurotol.

[13] Balkany TJ, Hodges AV, Buchman CA, Luxford WM, Pillsbury CH, Roland PS (2005). Cochlear implant soft failures consensus development conference statement. Otol Neurotol.

[14] Durand M, Michel G, Boyer J, Bordure P (2022). Auditory performance after cochlear reimplantation. Eur Ann Otorhinolaryngol Head Neck Dis.

[15] Costa LBA (2018). Avaliação da percepção auditiva da fala em pacientes submetidos ao reimplante coclear.

[16] Manrique-Huarte R, Huarte A, Manrique MJ (2016). Surgical findings and auditory performance after cochlear implant revision surgery. Eur Arch Otorhinolaryngol.

[17] Sterkers F, Merklen F, Piron JP, Vieu A, Venail F, Uziel A (2015). Outcomes after cochlear reimplantation in children. Int J Pediatr Otorhinolaryngol.

[18] Kim C-S, Kim D-K, Suh M-W, Oh SH, Chang SO (2008). Clinical outcomes of cochlear reimplantation due to device failure. Clin Exp Otorhinolaryngol.

[19] Mahtani S, Glynn F, Mawman DJ, O’Driscoll MP, Green K, Bruce I (2014). Outcomes of cochlear reimplantation in adults. Otol Neurotol.

[20] Seyyedi M, Nadol JB (2014). Intracochlear inflammatory response to cochlear implant electrodes in humans. Otol Neurotol.

[21] Havenith S, Lammers MJW, Tange RA, Trabalzini F, della Volpe A, van der Heijden GJMG (2013). Hearing preservation surgery: cochleostomy or round window approach? A systematic review. Otol Neurotol.

[22] Santa Maria PL, Gluth MB, Yuan Y, Atlas MD, Blevins NH (2014). Hearing preservation surgery for cochlear implantation: a meta-analysis. Otol Neurotol.

[23] Di Maio S, Malebranche AD, Westerberg B, Akagami R (2011). Hearing preservation after microsurgical resection of large vestibular schwannomas. Neurosurgery.

[24] Clark GM, Clark J, Cardamone T, Clarke M, Nielsen P, Jones R (2014). Biomedical studies on temporal bones of the first multi-channel cochlear implant patient at the University of Melbourne. Cochlear Implants Int.

[25] Öz O, De Ceulaer G, Govaerts PJ (2022). Speech audiometrical results before and after reimplantation of cochlear implants. Ear Hear.

[26] Birman CS, Sanli H, Gibson WPR, Elliott EJ (2014). Impedance, neural response telemetry, and speech perception outcomes after reimplantation of cochlear implants in children. Otol Neurotol.

[27] Kim C-S, Kim D-K, Suh M-W, Oh SH, Chang SO (2008). Clinical outcomes of cochlear reimplantation due to device failure. Clin Exp Otorhinolaryngol.

[28] Hamzavi J, Baumgartner WD, Pok SM (2002). Does cochlear reimplantation affect speech recognition?. Int J Audiol.

[29] Fayad JN, Eisenberg LS, Gillinger M, Winter M, Martinez AS, Luxford WM (2006). Clinical performance of children following revision surgery for a cochlear implant. Otolaryngol Head Neck Surg.

