# Supplementary material for: Analysis of aided thresholds in children who have undergone cochlear reimplantation: a ten-year follow-up
Source: Codas. 2023 Oct 30;35(6):e20210293. doi: 10.1590/2317-1782/20232021293en (PMC10702709; doi:10.1590/2317-1782/20232021293en)
Supplement: Translation

# Translation: Análise de limiares tonais de crianças submetidas ao reimplante: seguimento de 10 anos

**DOI:** 10.1590/2317-1782/20232021293pt

**Authors:** Lucas Bevilacqua Alves da Costa, Leticia Cristina Vicente, Leandra Tabanez do Nascimento Silva, Kátia Freitas Alvarenga, Manoel Henrique Salgado, Orozimbo Alves Costa, Rubens Brito

**Affiliations:** 1 Alfa Instituto de Comunicação e Audição - São Paulo (SP), Brasil.; 2 Centro Universitário Planalto do Distrito Federal - Brasília (DF), Brasil.; 3 Seção de Implante Coclear, Hospital de Reabilitação de Anomalias Craniofaciais (HRAC) - Bauru (SP), Brasil.; 4 Departamento de Fonoaudiologia, Faculdade de Odontologia - FOB, Universidade de São Paulo - USP - Bauru (SP), Brasil.; 5 Departamento de Engenharia de Produção, Faculdade de Engenharia de Bauru, Universidade Estadual Paulista - UNESP - Bauru (SP), Brasil.; 6 Departamento de Otorrinolaringologia, Faculdade de Medicina, Universidade de São Paulo - USP - São Paulo (SP), Brasil.

**Keywords:** Criança, Reimplante, Implante Coclear, Audição, Audiometria de Tons Puros

## Abstract

**Objetivo:**

Caracterizar os limiares auditivos nas frequências de 500, 1000, 2000 e 4000 Hz em crianças submetidas ao reimplante com follow-up de no mínimo 10 anos.

**Método:**

Revisão retrospectiva de prontuários de crianças submetidas a cirurgia de reimplante há pelo menos 10 anos. Foram avaliados os limiares auditivos obtidos na audiometria tonal liminar em campo livre com o implante coclear nas frequências de 500, 1000, 2000 e 4000 Hz em quatro momentos distintos: 1 (antes da falha), 2 (ativação), 3 (cinco anos após o reimplante) e 4 (dez anos após o reimplante, independentemente do tempo de uso do uso do 2o IC) em pacientes com follow-up de no mínimo 10 anos.

**Resultados:**

Avaliando-se pacientes submetidos ao reimplante, observou-se que os limiares de 500, 1000, 2000, 4000 Hz apresentaram-se a longo prazo semelhante àqueles obtidos nos pacientes implantados somente uma única vez, não apresentando prejuízo assim na detecção dos sons.

**Conclusão:**

O reimplante não teve efeito de longo prazo sobre os limiares auditivos obtidos em crianças que se submeteram a esta cirurgia por falha do componente interno.

## INTRODUÇÃO

O implante coclear tem sido realizado rotineiramente, estima-se que há cerca de 500.000 implantados em todo o mundo, o procedimento cirúrgico é considerado seguro e bem-sucedido para o tratamento das perdas auditivas severas a profundas^(1)^. Sendo assim, é o principal meio para a Reabilitação e Habilitação auditiva de tais casos, possibilitando às crianças o desenvolvimento da audição e linguagem, melhor desempenho escolar, além de melhores perspectivas quanto à futura inserção no mercado de trabalho^(2-4)^. Em relação aos adultos pós-linguais, o implante coclear é capaz de reestabelecer a comunicação e melhorar a qualidade de vida^(5-7)^.

O fato de as primeiras cirurgias de implante coclear multicanal terem sido realizadas na década de 90 gera uma preocupação natural entre os profissionais da área se haverá um momento em que o componente interno deixará de funcionar adequadamente pelo desgaste do tempo e consequentemente exigirá uma nova intervenção cirúrgica. Estudos demonstram que a taxa de revisão cirúrgica do implante coclear está entre 5 a 10%^(1)^, sendo que a causa mais frequente é a falha do dispositivo, sendo necessário o reimplante^(8)^.

Além dos riscos inerentes à intervenção cirúrgica, especificamente no reimplante existe a preocupação com o impacto no desempenho audiológico e na percepção auditiva da fala, ou seja, se o paciente manterá o benefício obtido com a primeira cirurgia. Essa questão é relacionada à possível dificuldade que o cirurgião poderá ter ao inserir um novo eletrodo na cóclea, podendo este fazer um trajeto diferente do antigo eletrodo, estimulando por sua vez outras porções da cóclea^(9)^. Além disso, há a possibilidade da cóclea estar ossificada, impedindo a retirada do antigo eletrodo ou esta retirada pode ser parcial, o que invariavelmente irá afetar o desempenho auditivo do paciente^(9)^.

A análise dos resultados obtidos após a cirurgia de reimplante por diversas causas têm contribuído para aumentar o conhecimento das consequências da nova intervenção cirúrgica^(10)^. Dentre as causas descritas para o reimplante, estão a *Hard Failure* que é caracterizada por uma parada abrupta do funcionamento do componente interno do implante coclear e a *Soft Failure* que é caracterizada por uma queda no desempenho audiológico do paciente, por vezes essa queda pode ser progressiva e confundida com questões do desenvolvimento da criança que podem estar associadas à comorbidades e questões cognitivas, essa falha é de difícil detecção, em muitos casos é possível verificar alterações da telemetria de impedância e neurotelemetria^(11-13)^. A *Hard Failure* é descrita como a causa mais comum que leva ao reimplante^(14-18)^.

No geral, o reimplante é considerado um procedimento seguro, com bons resultados auditivos na maioria dos pacientes^(9,14,15,17-19)^ . No entanto, entre os estudos, não há estudos longitudinais de longo prazo para descrever se os resultados encontrados em crianças relacionados aos limiares auditivos são mantidos muitos anos após o reimplante. Tais informações são essenciais uma vez que a atenção e detecção auditiva são habilidades auditivas necessárias para o desenvolvimento de habilidades mais complexas, como a percepção de fala.

Tendo em vista que a atenção auditiva e a detecção são as primeiras habilidades auditivas a serem adquiridas pelas crianças e são fundamentais para o processo de desenvolvimento das demais, o objetivo deste estudo foi caracterizar os limiares auditivos ao longo de 10 anos em crianças submetidas ao reimplante coclear.

## MÉTODO

O presente estudo foi aprovado pelo Comitê de Ética em Pesquisa do Hospital de Reabilitação de Anomalias Craniofaciais da Universidade de São Paulo (HRAC-USP) sob o número 673836/2014. Trata-se de um estudo retrospectivo e longitudinal realizado por meio da análise de prontuários de indivíduos atendidos na Seção de Implante Coclear do Centro de Pesquisas Audiológicas do HRAC-USP. No total de 1323 cirurgias de implante coclear realizadas no período de 1990 a janeiro de 2016, consta o registro de 84 cirurgias de reimplante cocleares, correspondendo a 6,3% do total.

Foram selecionados dados de 12 usuários de implante coclear unilateral que apresentaram *hard failure* do dispositivo interno Combi 40+ da MED-EL e que foram submetidos ao reimplante coclear com outro dispositivo interno Combi 40+ na mesma orelha. Todos apresentavam inserção total dos eletrodos, realizaram acompanhamento do desenvolvimento das habilidades auditivas antes da falha e por um período de dez anos após a cirurgia de reimplante, além disso, não possuíam quaisquer comprometimentos neurológicos e/ou cognitivos. A descrição da idade dos participantes na primeira cirurgia de implante coclear (IC), no reimplante e o tempo de reabordagem estão descritos na Tabela 1.

**Tabela 1:** Dados Demográficos

Criança	Etiologia	Idade 1ª cirurgia (meses)	Tempo de uso do IC na falha do dispositivo (meses)	Orelha Implantada	Tempo Reabordagem (meses)
1	Citomegalovírus	32	19	OD	2
2	Idiopática	45	25	OD	3
3	Rubéola	89	66	OE	1
4	Idiopática	46	37	OE	4
5	Idiopática	37	22	OE	4
6	Idiopática	41	20	OE	2
7	Ototóxico	55	46	OD	5
8	Rubéola	84	44	OD	2
9	Meningite	62	30	OD	3
10	Meningite	24	31	OE	4
11	Idiopática	40	46	OE	1
12	Meningite	36	32	OD	5
Média ± DP	-	49,25±20,06	34,83±13,78		3±1,41
Meses		(32-89)	(19-66)		(1-5)

Legenda: DP = Desvio Padrão; IC = Implante Coclear; OD = Orelha Direita; OE = Orelha Esquerda

### Procedimentos

Foram avaliados os limiares auditivos obtidos na audiometria tonal liminar em campo livre somente com o uso do implante coclear nas frequências de 500, 1000, 2000 e 4000 Hz em quatro momentos distintos: 1 (antes da falha), 2 (ativação), 3 (cinco anos após o reimplante) e 4 (dez anos após o reimplante, independentemente do tempo de uso do uso do 2º IC).

### Análise dos dados

A análise estatística descritiva dos limiares auditivos foi realizada considerando as frequências testadas de 500, 1000, 2000 e 4000 Hz nos quatro momentos de avaliação.

As correlações entre as frequências testadas e momentos de avaliação foram realizadas por meio da Two-way ANOVA. Os testes Post hoc, Tukey method foram utilizados para calcular as diferenças significantes entre os momentos de avaliação. O nível de significância estatística foi estabelecido em 5%.

## RESULTADOS

A Tabela 2 fornece informações sobre média, faixa, desvio padrão e mediana dos limiares auditivos em 500, 1000, 2000 e 4000 Hz medidos antes da falha do dispositivo, na ativação do dispositivo reimplantado e após 5 e 10 anos após o reimplante.

**Tabela 2:** Descrição dos limiares auditivos obtidos nas frequências de 500, 1000, 2000 e 4000 Hz obtidos nas avaliações 1 (antes da falha), 2 (ativação), 3 (cinco anos após o reimplante) e 4 (dez anos após o reimplante)

**Frequência** **(Hz)**	**Momentos de avaliação**	**Limiares auditivos (dB NA)**				
		**Média**	**DP**	**Min**	**Max**	**Mediana**
500	Antes da Falha	33,33	4,92	25,00	40,00	35,00
	Ativação	37,92	9,64	30,00	65,00	35,00
	Após 5 anos	23,33	4,92	20,00	35,00	20,00
	Após 10 anos	24,17	5,15	20,00	35,00	22,50
						
1000	Antes da Falha	35,83	4,69	30,00	45,00	35,00
	Ativação	37,92	9,16	25,00	60,00	35,00
	Após 5 anos	24,58	4,50	20,00	30,00	25,00
	Após 10 anos	25,00	4,77	20,00	35,00	25,00
						
2000	Antes da Falha	33,75	5,28	25,00	40,00	35,00
	Ativação	41,67	11,15	30,00	60,00	40,00
	Após 5 anos	23,75	4,33	20,00	30,00	22,50
	Após 10 anos	24,58	6,20	20,00	40,00	22,50
						
4000	Antes da Falha	35,42	6,89	25,00	45,00	35,00
	Ativação	42,08	12,33	30,00	65,00	37,50
	Após 5 anos	26,25	5,28	20,00	35,00	27,50
	Após 10 anos	27,92	6,20	20,00	35,00	27,50

Legenda: DP = Desvio Padrão; Min = Mínimo; Max = Máximo

Comparações entre os limiares medidos antes da falha do IC e nas sessões seguintes revelaram que embora algumas crianças apresentassem limiares piores logo após a ativação, todos os limiares obtidos no seguimento de cinco e dez anos foram mantidos ou melhorados. A diferença dos limiares entre as sessões antes e após o reimplante considerados como melhora ou piora são apresentados na Figura 1.

**Figura 1:** A média ± erro padrão (EP) para diferenças de limiares consideradas como uma melhora ou piora para 500, 1000, 2000 e 4000 Hz é plotada para cada comparação antes e após o reimplante coclear. O número de crianças considerado para cada valor é apresentado acima do símbolo de média. Os resultados das demais crianças não foram considerados neste gráfico, uma vez que seus limiares se mantiveram após o reimplante, ou seja, a diferença foi de 0 ou 5 dB

A ANOVA bidirecional para medidas repetidas foi utilizada para comparar os limiares auditivos de acordo com a frequência e o tempo de medição. Observou-se efeito principal apenas para o tempo de medição, não havendo interação significativa com as frequências testadas (Tabela 3). As médias dos limiares para cada frequência foram calculadas em média para análise post-hoc. Um teste post-hoc de Tukey não revelou nenhuma diferença significativa entre os limiares em 5 e 10 anos após o reimplante. No entanto, esses limites são significativamente melhores do que os medidos antes da falha do dispositivo e na ativação. Os valores dos limiares foram piores na ativação do que os obtidos antes da falha (Figura 2).

**Tabela 3:** Comparação dos limiares auditivos entre os momentos de avaliação e frequências testadas (Two-way ANOVA)

	**Frequências**	**p**
Momentos	53,54	0,000*
Frequência	1,74	0,160
Interação	0,33	0,963

* Nível de significância p≤0,05

**Figura 2:** Média ± erro padrão (EP) em todas as frequências testadas (500, 1000, 2000 e 4000 Hz) é traçado para cada tempo de medição. Letras diferentes acima das barras indicam diferenças significativas no teste de Tukey (p <0,05)

## DISCUSSÃO

Em relação ao reimplante coclear, a necessidade de realização da retirada seguida de nova inserção do arranjo de eletrodos intracocleares levanta a questão se esta intervenção poderia comprometer os resultados audiológicos. Seyyedi e Nadol^(20)^ observaram achados temporais de 21 adultos usuários de IC. Um processo inflamatório crônico nos eletrodos levou à formação de fibrose e nova formação de osso, a resposta inflamatória foi menos intensa ao redor dos eletrodos mais próximos da região mais apical da cóclea. É possível que tal condição dificulte a inserção de eletrodos em um eventual reimplante. A preocupação com o trauma de inserção do eletrodo no IC levou ao conceito de *soft surgery*. Na *soft surgery*, a abordagem cirúrgica visa preservar a estrutura da cóclea pela combinação de diversos procedimentos, como o uso de substâncias associadas à preservação auditiva: ácido hialurônico e corticóides, eletrodos menos invasivos e um tempo maior na inserção do eletrodo, tais práticas são importantes visando não só a preservação de resíduos auditivos, mas também melhores condições cirúrgicas em caso de necessidade de reimplante^(21-23)^.

Além da condição da cóclea no momento do reimplante, é bom ter em mente que em uma nova inserção do feixe de eletrodos, nem sempre este seguirá a trilha primária^(10)^. Assim, o novo arranjo de eletrodos pode estar localizado em um espaço intracoclear diferente do primeiro inserido, o que permite questionar qual seria o impacto de tal alteração no desempenho auditivo, uma vez que porções diferentes da cóclea poderão ser estimuladas. Clark et al.^(24)^, descreveram a trajetória de um paciente que foi reimplantado duas vezes e, após seu óbito, os achados histopatológicos do osso temporal indicaram perda total do órgão de Corti em ambos os lados e ruptura da membrana basilar do lado esquerdo.

No presente estudo, a avaliação dos limiares auditivos em crianças submetidas ao reimplante demonstrou que os limiares obtidos na ativação são piores em comparação aos medidos antes do reimplante, ou seja, os indivíduos não apresentaram o mesmo desempenho na detecção logo após a segunda cirurgia (Figura 2). Vale ressaltar que o tempo de reabordagem entre a falha do dispositivo e o reimplante não ultrapassou o período de 5 meses, sendo realizado em média no período de 3 meses, portanto os pacientes não tiveram longo período de privação auditiva até o momento do reimplante. Os acompanhamentos de cinco e dez anos dos limiares auditivos após o reimplante mostraram que a piora imediata dos limiares após a ativação foi temporária e pode estar relacionada a um menor nível de corrente disponibilizado no momento da ativação, visando maior conforto e uso efetivo do dispositivo. Os limiares se recuperaram e não houve indivíduos com limiares piores que 5 dB após o reimplante em relação aos verificados na última audiometria tonal antes da falha do aparelho. De fato, os limiares após cinco e dez anos de reimplante foram ainda melhores que os anteriores (Figura 1 e Figura 2), demonstrando que o reimplante não prejudicou o desenvolvimento das habilidades auditivas.

Embora a frequência não tenha tido um efeito significativo na mudança dos limiares (Tabela 3), foram observados limiares auditivos piores que 40 dB NA na ativação em 16,66% das crianças para as frequências de 500 e 1000 Hz, enquanto para as frequências mais altas (2000 e 4000 Hz) a incidência dobrou (33,33%). Os eletrodos do IC são colocados para manter a organização tonotópica da cóclea, portanto, informações de alta frequência são entregues aos eletrodos na região basal. Como essa região tem maior probabilidade de sofrer trauma cirúrgico^(24)^, limiares auditivos elevados logo após o reimplante podem ser piores do que aqueles medidos em frequências mais baixas. Estudos com mais participantes devem ser realizados para verificar essa hipótese. No entanto, os resultados obtidos em cinco e dez anos de acompanhamento demonstraram que a funcionalidade de detecção é mantida mesmo para altas frequências.

Neste estudo, houve a possibilidade de analisar longitudinalmente os dados audiológicos de longo período em usuários de IC que foram reimplantados com o mesmo tipo de IC e apresentavam a mesma causa de reimplante, *Hard Failure* do dispositivo. O controle dessas variáveis permitiu concluir que o protocolo de acompanhamento dos pacientes após o reimplante não requer modificações, ou seja, deve ser realizado o mesmo protocolo de acompanhamento do usuário de implante coclear sem reimplante, consistindo em retornos periódicos para mapeamento, manutenção adequada do aparelho, avaliação do desenvolvimento auditivo e de linguagem e atualização do processador quando necessário.

Outro achado significativo foi que, o bom resultado relacionado aos limiares auditivos alcançados cinco anos após o reimplante foram mantidos nos cinco anos posteriores (Figura 1).

O paciente que necessita de reimplante e a família devem ser informados de que embora essa cirurgia seja considerada um procedimento seguro na maioria dos casos, bons resultados não são garantidos. Uma diminuição no desempenho auditivo deve ser esperada logo após o reimplante, mas os limiares auditivos provavelmente serão recuperados e se manterão com bons níveis até dez anos após o reimplante. No entanto, embora seja um percentual pequeno, há relatos de pacientes que não recuperam o desempenho anterior ao reimplante^(15,25,26)^.

Muitos estudos focam na percepção da fala após o reimplante^(15,17,25,27,28)^, visto que esta é a habilidade alvo com o IC. No entanto, dada a complexidade de tal habilidade e a idade dos participantes deste estudo, optou-se por analisar a habilidade primordial para se atingir as demais, ou seja, a detecção. Além disso, tal habilidade sofre menor influência de aspectos relacionados ao próprio desenvolvimento da criança, portanto, em uma análise pré e pós intervenção os limiares auditivos demonstram ser um bom parâmetro do impacto que o reimplante pode causar na audição do paciente com o uso do IC. Os achados deste estudo corroboram com pesquisa que demonstrou que após o reimplante de 18 crianças, não foram encontradas diferenças estatisticamente significantes entre os limiares tonais pré e pós reimplante^(29)^.

## CONCLUSÃO

Os limiares obtidos nas frequências de 500, 1000, 2000 e 4000 Hz em 10 anos de uso do IC após o reimplante mostraram que pode haver uma piora imediata constatada na ativação após a segunda cirurgia, mas que se recuperam na análise de cinco anos de uso. No período posterior de cinco para dez anos os limiares mantêm-se estáveis.
